# Augmenting Image-Guided Procedures through In Situ Visualization of 3D Ultrasound via a Head-Mounted Display

**DOI:** 10.3390/s23042168

**Published:** 2023-02-15

**Authors:** Felix von Haxthausen, Christoph Rüger, Malte Maria Sieren, Roman Kloeckner, Floris Ernst

**Affiliations:** 1 Institute for Robotics and Cognitive Systems, University of Lübeck, 23562 Lübeck, Germany; 2 Department of Surgery, Campus Charité Mitte, Campus Virchow-Klinikum, Experimental Surgery, Charité–Universitätsmedizin Berlin, Corporate Member of Freie Universität Berlin, Humboldt-Universität zu Berlin, Berlin Institute of Health, 10117 Berlin, Germany; 3 Department of Radiology and Nuclear Medicine, University Hospital Schleswig-Holstein Campus Lübeck, 23569 Lübeck, Germany; 4 Institute of Interventional Radiology, University Hospital Schleswig-Holstein Campus Lübeck, 23569 Lübeck, Germany

**Keywords:** augmented reality, HoloLens 2, volume rendering, ultrasound phantom, mixed reality

## Abstract

Medical ultrasound (US) is a commonly used modality for image-guided procedures. Recent research systems providing an in situ visualization of 2D US images via an augmented reality (AR) head-mounted display (HMD) were shown to be advantageous over conventional imaging through reduced task completion times and improved accuracy. In this work, we continue in the direction of recent developments by describing the first AR HMD application visualizing real-time volumetric (3D) US in situ for guiding vascular punctures. We evaluated the application on a technical level as well as in a mixed-methods user study with a qualitative prestudy and a quantitative main study, simulating a vascular puncture. Participants completed the puncture task significantly faster when using 3D US AR mode compared to 2D US AR, with a decrease of 28.4% in time. However, no significant differences were observed regarding the success rate of vascular puncture (2D US AR—50% vs. 3D US AR—72%). On the technical side, the system offers a low latency of 49.90 ± 12.92 ms and a satisfactory frame rate of 60 Hz. Our work shows the feasibility of a system that visualizes real-time 3D US data via an AR HMD, and our experiments show, furthermore, that this may offer additional benefits in US-guided tasks (i.e., reduced task completion time) over 2D US images viewed in AR by offering a vividly volumetric visualization.

## 1. Introduction

Medical ultrasound (US) imaging has become an indispensable technology for diagnosis and intervention in clinical practice. The technique is widely available and offers significant advantages over other imaging modalities such as computed tomography (CT) and magnetic resonance imaging (MRI) due to its safeness, mobility, cost-effectiveness, and real-time imaging of moving organ structures. Nevertheless, this technology suffers from several disadvantages. First, mentally aligning the 2D cross-sectional US image with the 3D anatomy requires skill and experience. Second, out-of-plane motion due to breathing movement or similar results in imaging of different undesired anatomical slices. Third, the relocation of the probe at the exact same position between different examinations, as needed for monitoring the progression of a pathology, is difficult. Moreover, the physician’s gaze is focused on the US system, not being able to perceive the spatial relationship between the pose of the probe, the scanned anatomical region and its surroundings, e.g., interventional instruments.

Especially for US-guided needle interventions, augmented reality (AR)-based in situ visualization of the image may be beneficial as it shows the structure to be hit in its actual physical location. AR systems based on head-mounted displays (HMDs) can be particularly beneficial because they provide the user with an egocentric perception of the environment, a stereoscopic display of virtual elements, and additionally facilitate hands-free interaction. Recent user studies have investigated the combination of 2D US with AR-HMDs for needle guidance tasks with respect to conventional US. It was shown that the combination leads to reduced task completion times [1,2] and improved accuracy [1,3,4]. Such benefits are likely due to improved hand–eye coordination as well as spatial awareness and understanding [3]. Even AR systems using a tablet for visualization proved significant performance improvements of novices in both objective and subjective evaluation metrics after training with the application [5]. In order to guide prostate biopsy procedures in a biopsy simulator, Cool et al. [6] compared the accuracy of 2D transrectal US with that of conventional 3D transrectal US and guided 3D transrectal US, where the latter included segmented biopsy targets. The accuracy of the biopsy performed by both specialists and residents increased when using guided 3D transrectal US compared to 2D transrectal US, but not when using standard 3D transrectal US. However, only orthogonal views, and no volume-rendered image, were displayed that might add additional spatial knowledge. Moreover, no in situ visualization was provided by the system.

To overcome the remaining disadvantages displayed by US (out-of-plane motion, difficulties in placing the probe to view both needle and target anatomy), this work continues in the direction of recent developments in AR US: We propose the in situ visualization of real-time 3D US using an HMD for needle guidance purposes to further improve image guidance. Since 2D US with AR has been shown to be advantageous over conventional 2D US, we refrain from comparing it with conventional US in this work and solely compare the combination of 3D US with AR versus 2D US with AR. To test initial feasibility of the setup, we first conducted a qualitative study to gather feedback. Based on the findings of this prestudy, we chose a US-guided vascular puncture task as a representative use case. In this context, we hypothesize that 3D US with AR leads to improved rates of success and reduced task duration due to improved image guidance offered by volumetric images. As a secondary endpoint, we measure the perceived task load for both combinations with the assumption of a lower task load with the 3D US AR setup compared to the 2D US AR setup.

To this end, we followed a mixed-methods approach, consisting of a qualitative prestudy followed by a quantitative main study. The conclusions of the prestudy were used to improve the HMD application and to design and implement the main study, which in turn serves to test the hypotheses. Furthermore, the methodically collected subjective assessments by experts within the prestudy do provide important information regarding the clinical applicability of the overall system, which is of interest for other researchers in this field. We also performed a brief technical evaluation, including latency of visualized images and frame rate of our application.

### Related Work—HMD-Based 3D Ultrasound Guided Interventions

Two recent studies [7,8] showcased a similar system, namely, an AR HMD navigation system with a 3D US imaging system to guide surgical interventions. In [8], an in vitro study simulating a biopsy intervention showed that 80% of participants were able to perform a biopsy on a 5 mm lesion. In [7], the platform was extended by a tracked standard scalpel to provide real-time information on the pose of the instrument. An in vitro experiment showed that the system can be used to guide a dissection task with a mean accuracy of 0.65 mm. Even though both studies showed promising results, the systems did not visualize the live volumetric US image, but instead a segmented structure from a previously acquired US volume. Therefore, any potential positional shift of the target anatomy may result in misleading visual guidance. In this work, on the other hand, the system streams and visualizes live volumetric images from a commercially available US station. Accordingly, any change in position or orientation of the target anatomy becomes immediately visible.

So far, technical setups for visualizing sonography in spatially-aligned real-time AR have been limited to 2D images. Accordingly, our main contributions to the field are:

1. A novel technical setup for visualizing volumetrically 3D US through an AR HMD;
2. A demonstration that this type of setup can offer benefits in image-guided tasks;
3. To show that 3D/volumetric data can offer additional benefits in spatial understanding over 2D data in this context.

At the same time, we present the difficulty of clearly visualizing relevant structures when surrounded by occluding objects and highlight the need to address this issue.

## 2. Materials and Methods

### 2.1. System Description

**Figure 1:**
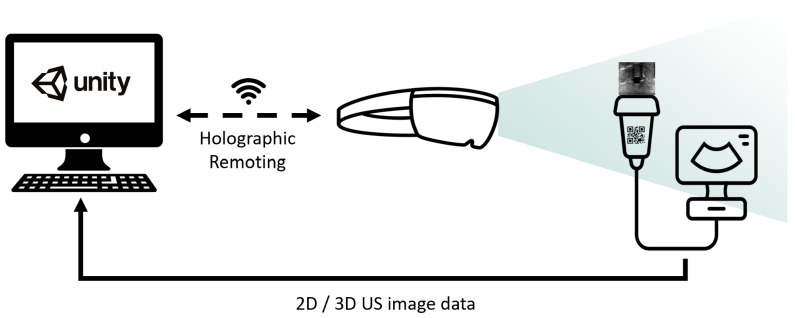
The general technical setup, consisting of remote rendering workstation running the Unity application, the HoloLens 2 displaying the rendered images while tracking the US probe, and the US station sending images or volumes to the workstation. Solid lines indicate an Ethernet connection and dashed ones wireless connections.

The centerpiece of the system is an HMD incorporating a self-contained computer and an optical see-through display. This HMD provides the visualization of intraprocedural image data and performs US probe tracking (Figure 1). We chose the HoloLens 2 (Microsoft, Redmond, WA, USA) for the following features: hand gestures and voice commands for interaction, a remote rendering option [9], low weight, and the stereoscopic display to provide a 3D perception of volumetric objects.

The HoloLens 2 application was developed with Unity 2020.3.7f1 (Unity Technologies, San Francisco, CA, USA). Instead of relying on the HoloLens’ onboard hardware, which might be insufficient for computationally expensive real-time volume rendering, we utilized the holographic remoting mode (HRM) [9]. This mode enables remote rendering on a capable workstation, streaming the rendered images to the HMD while the user is wearing them. The remote rendering workstation in this experiment was equipped with an Intel Xeon(R) E5-2697 v4 CPU, an NVIDIA Quadro M6000 GPU, and 64 gigabytes of RAM. Consequently, even computationally expensive stereoscopic volume rendering could potentially be performed at a target frame rate of 60 Hz. To visualize US images and volumes in situ, i.e., attached to the US probe, the probe must be tracked spatially. To minimize technical complexity, we preferred an inside-out tracking approach compatible with the HRM by using the built-in QR code tracking [10]. As the HRM does not provide access to the sensor data, we could not utilize a previously published tracking approach using retroreflective spheres [11]. For US data acquisition, we used an EPIQ 7 station (Philips Medical Systems International B.V., Best, The Netherlands) that allows real-time streaming of image data to the computer via a proprietary network protocol provided by Philips.

### 2.2. Ultrasound Image Data Streaming and Volume Rendering

For the qualitative prestudy, which was not limited to a specific use case, the X6-1 3D US probe was utilized. The quantitative main study, on the other hand, was performed with a novel 3D probe (XL14-3) that has a linear matrix array consisting of 65,000 elements. Therefore, it features the advantages of a linear probe (same line density at depth including high lateral resolution) while at the same time being able to acquire a real-time volumetric US image.

Eight-bit 2D and 3D image data were transmitted in real-time from the US station to the Unity instance running on the computer using the proprietary network protocol. To perform volume rendering (GPU-based raycasting in this case), the choice of an appropriate opacity transfer function (OTF) is needed [12]. This transfer function maps the intensity value *I* of each voxel to an according opacity value α. A simple piecewise linear OTF is sufficient for visualization of structures embedded in areas with low signal intensity [13]. A US volume showcasing vascular structures in a homogeneous hypoechoic tissue can be considered as such a use case. Therefore, the OTF for this application is defined as

(1) $$\alpha \left(I\right)=\left\{\begin{array}{cc} 0, & \mathrm{if}\;I<L_{l}\\ a(I-L_{l}), & \mathrm{if}\;L_{l}<I<L_{u}\\ a(L_{u}-L_{l}), & \mathrm{if}\;I>L_{u} \end{array}\right.$$

where $L_{l}$ is the lower limit, $L_{u}$ is the upper limit, and a∈[0,1] is a scaling factor. The US settings (including volume rendering parameters) were prepared by supervisors before the start of the participant’s task to optimally visualize anatomical structures in 2D and 3D images. The settings are summarized in Table 1.

### 2.3. Spatial Calibration for Tracked Ultrasound Probe

In this work, the marker had a size of 6 × 6 cm, which was the minimum size to reliably track the code at an arm’s length distance. To increase rotational movement freedom and based on the feedback of the prestudy, the probe was equipped with a second QR code. Therefore, even if line-of-sight is not guaranteed for the first QR code, the pose of the probe is still known due to the second tracked QR code. According to [10], the QR code can be detected up to an angle of ±45°. Assuming that one can just add the rotational shift of the second QR code with respect to the first one (50°), our adapted system offers a detection range of up to ±70°. The calibration was performed for one marker only. Having the relative transformation between the two codes enables them to extend the calibration to the second one.

The attached QR code provides the current position and orientation of the US probe with respect to the HoloLens coordinate system. To visualize the US image based on the QR code pose in situ, it is necessary to find the spatial transformation from the QR code origin to the US image coordinate system by means of a spatial calibration. Our approach is based on point cloud matching of geometrical shapes (spheres and cylinders) within a commercial US calibration phantom (CIRS Inc., Norfolk, VI, USA).

**Figure 2:**
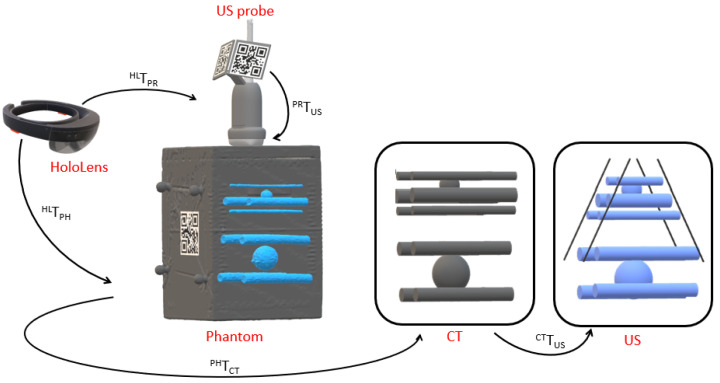
Transformations (black arrows) between the different coordinate systems (red) necessary for probe calibration. A multimodality calibration phantom containing spheres and cylinders is used for the point-cloud-based registration approach.

In order to visualize a voxel v (or pixel for 2D) of an US image in situ, the following equation is required:

(2) vHL=THLPRTPRUSmmAUSmmUSvUS

where va is a voxel in coordinate system *a*, Tab∈R4×4 is a homogeneous rigid transformation that maps a point from coordinate system *b* to coordinate system *a*, and Aab∈R4×4 is an affine transformation between coordinate system *b* and *a*. The coordinate systems HL, PR, USmm, and US refer to HoloLens, US probe, volume in mm, and volume, respectively. The unknown transformation TPRUSmm can be calculated by closing the transformation loop as illustrated in Figure 2 and by using the following equation:

(3) TPRUSmm=(THLPR)−1THLPHTPHCTTCTUSTUSUSmm

where the coordinate systems PH and CT refer to phantom and CT, respectively. In order to calculate TPRUSmm, the two missing transformations TPHCT and TCTUS have to be determined. A landmark-based registration provides TPHCT, as the QR code is placed on the phantom surface in a known position, and the corner positions can be defined both in the CT and HoloLens coordinate system. On the other hand, a point cloud matching of the segmented geometrical shapes of the phantom within the CT and US coordinate system can be used for calculating TCTUS. Point clouds from CT and US data are created by applying a Canny edge detection to the volumes. Having both point clouds and an initial prealignment, the iterative closest point algorithm (ICP) is applied to find TCTUS with 200 randomly sampled points of each model. Further details on the calibration and its accuracy can be found in [14].

For displaying the 2D US image, the middle slice of the US volume was selected. Since the pose of the 2D plane in the volume is known, no further calibration was necessary to display the image in situ.

### 2.4. Phantom Fabrication

**Figure 3:**
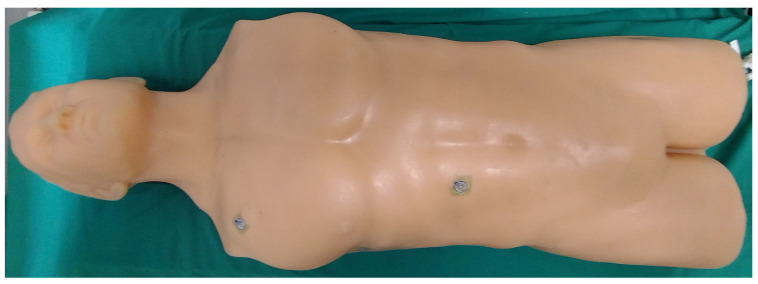
The US-capable torso phantom contains several organs and vessels and is therefore suitable for the exploratory purpose of the qualitative prestudy.

As the qualitative prestudy was not limited to any specific medical field, and due to its exploratory purpose, we used an anthropomorphic torso phantom (Figure 3, FAST Trauma Training Model, CAE Inc. Blue Phantom, Montreal, QC, Canada). This US-capable phantom contains several vessels and organs (e.g., pulmonary vessels, ascending aorta, liver, heart, spleen, bladder, and kidneys).

For the main study, phantoms were custom-made to fulfill the following requirements: US-capable, perfusable artificial vessels including a bifurcation, repairable, and a transparent tissue with an opaque skin layer. To avoid any bias from seeing previous insertion channels in the US image, which was also mentioned by a previous work [3], the repair function was essential. To this end, we chose ballistic gel (Clear Ballistics, Greenville, SC, USA) which allows remelting once heated [15,16,17].

**Figure 4:**
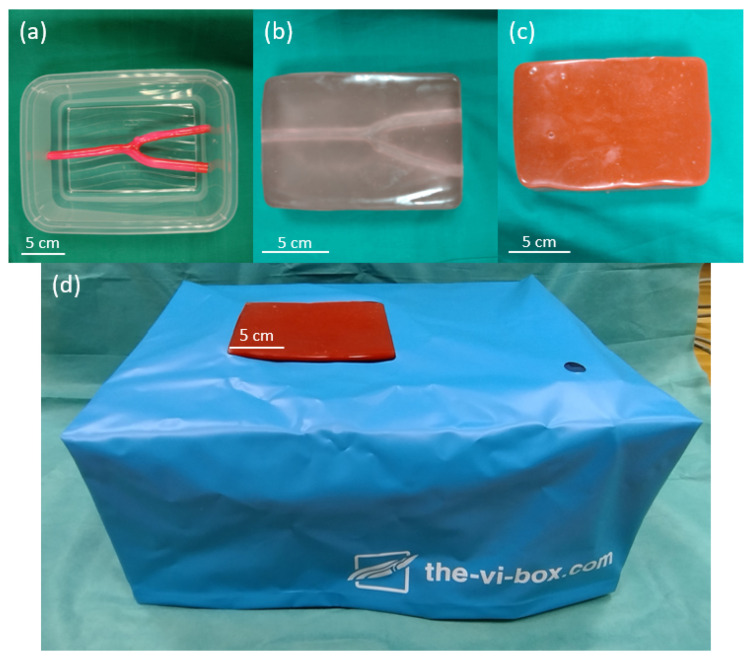
The vessel phantom for the main study. (**a**) The container including a silicone vessel with bifurcation before adding ballistic gel. (**b**) The gel phantom after removing it from the container and the vessel. (**c**) The opaque skin layer on top of the phantom prevents the participants from seeing the vessel. (**d**) The phantom placed inside the vi-box which contains a water reservoir and pumps to perfuse the artery with a pulsatile flow.

1.5 kg of gel were cut into small pieces, placed in an oven dish and heated in a kitchen oven at 125 °C for two hours. Thereafter, 2 w/w% paraffin wax (Bayerwald Brennstoffe GmbH, Arrach, Germany) and 0.5 w/w% solid glass spheres (∅ 0–63 µm, Boud Minerals, Lincolnshire, UK) were slowly added to increase acoustic attenuation and backscattering, respectively [18]. Hereafter, the mixture was put in the oven for another three hours at 125 °C to remove introduced air bubbles. The liquid gel with additives was then poured into a rectangular container (70 × 130 × 150 mm) with a silicone vessel including a bifurcation (Vascular International, Kerns, Switzerland) as illustrated in Figure 4a. It was then allowed to cool down for 24 h and the silicone vessel was removed by gently pulling it out (Figure 4b). To produce the skin layer, a brown liquid silicone dye was added to the remaining gel until it was opaque and then poured in a different container of the same size until a height of ∼5 mm (Figure 4c). In total, six phantoms were produced according to this procedure.

To perfuse the vessel with a pulsatile flow, the single end within the custom-made phantom is connected to the vi-box (Vascular International, Kerns, Switzerland). This device features a water reservoir and two small, battery-operated pumps which supply pulsatile or continuous flow. The custom-made phantom, the skin layer, and the vi-box form the setup for the quantitative main study (Figure 4d).

### 2.5. Evaluation

This research project followed a mixed-methods approach, combining a qualitative prestudy with a quantitative main study. With this exploratory sequential design, the results and conclusions of the prestudy were used to improve the application and inform the planning and execution of the main study. To acquire participants for both studies, we performed convenience sampling, resulting in eight participants for the prestudy and 18 for the main study.

#### 2.5.1. Qualitative Prestudy

The prestudy consisted of a think-aloud session followed by a semistructured interview (Supplementary Document S1: Prestudy protocol). First, we welcomed participants and introduced them to the context and agenda of the experiment, as well as acquiring consent. In the following think-aloud session, participants were asked to voice their thoughts while freely using the setup shown in Figure 1 on a US torso phantom (Figure 3), e.g., trying to locate and view various organs. Participants first had the opportunity to familiarize themselves with the US station, particularly its 3D mode, before beginning the session with the AR visualization. All participants started with conventional/2D US, followed by 3D US. After they decided that they had sufficiently tested each mode, the semistructured interview took place. The experiments’ audio was recorded and transcribed by the interviewer. The same person then performed a structuring qualitative content analysis (based on methods proposed by Kuckartz [19]) on the transcripts using Taguette [20]. Categories were initially formed deductively based on the existing literature [3] and then iterated upon inductively throughout the coding process, particularly the more granular subcategories for each major category. Segments were coded on a level of semantically closed and coherent phrases, usually encompassing a full sentence, but occasionally also multiple sentences. In addition to coding transcript segments into categories, summaries were written for each case/participant.

#### 2.5.2. Quantitative Main Study

As before, we welcomed participants, introduced them to the context of the experiment, and acquired the informed consent. Each participant was asked to puncture the vessel above the bifurcation with a needle (1.4 mm diameter, 17G, length 70 mm). The participants were supposed to perform the task one time with 2D US and the HMD and another time with 3D US and the HMD. The order was randomly chosen to minimize carryover effects. For each mode, a different phantom was used such that no insertion channels were visible. Note that the (usually echogenic) insertion channels were closed after each individual experiment by means of local heat using a soldering iron to prevent them from accumulating and interfering with later experiments.

Before the actual experiment, each participant was allowed to familiarize themself with the in situ visualization for 2D and 3D images by scanning the calibration phantom (Figure 2) without a time limit. As this phantom contains cylinders, the users had the chance to experience the visualization of vessel-like structures with the 2D and 3D mode.

**Figure 5:**
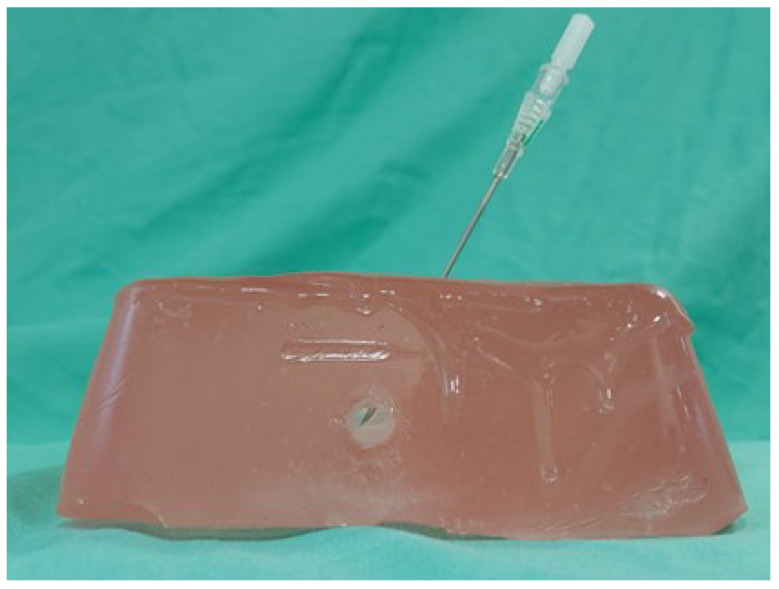
Photograph of the phantom with a placed needle to illustrate the easily identifiable successful puncture.

The primary endpoints were task duration (time to identify the bifurcation and placing the needle) and successful puncture of the vessel above the bifurcation. To this end, the time was taken from the initial placement of the probe on the phantom until the participant signaled that the task was finished. A visual inspection of the transparent phantom by the supervisor allowed us to verify if the vessel was successfully punctured (Figure 5). After both attempts, the participant was then asked to fill out two NASA task load index (TLX) questionnaires [21], once for each modality.

Statistical analysis was performed in Python (version 3.7.4 for Windows), including the scientific utility package *pingouin* (0.5.0) to perform hypothesis tests ($\alpha_{total}=5\%$, individual test α include Bonferroni–Holm corrections for multiple testing). The code and data are available as Supplementary Materials. The code for the analyses as well as a list of required Python packages (including exact versions) can be found in the Supplementary Materials S2: Evaluation code.

#### 2.5.3. Technical Evaluation

**Figure 6:**
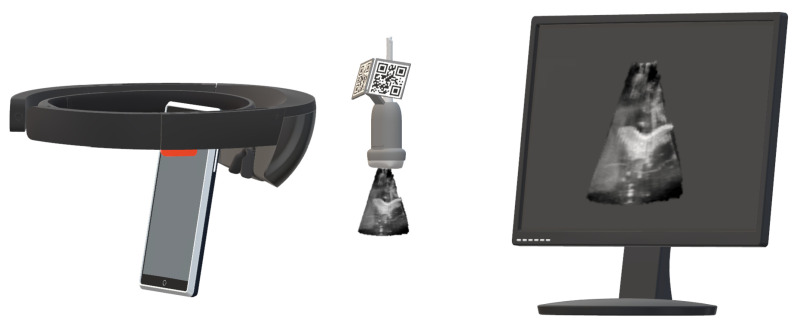
The schematic setup for measuring the latency of displayed US volumes. A slow-motion video captures the US station screen and the rendered US volume.

Low latency of visualized US volumes and images is crucial for the proposed use-case as advancing the needle towards a critical structure should be shown in near-real time. In this setup, we measure the latency by recording a slow motion video through one lens of HoloLens 2 while capturing the rendered volume on the lens and on the US station screen (Figure 6). During the recording, the depth of the US image was changed frequently. With a recording frequency of 240 Hz, a temporal resolution of 4.17 ms was achieved. For the actual measurement, the frames were counted between the visible change on the US station and the virtual US volume. The latency is, therefore, the delay between US station visualization and HMD visualization.

The target frame rate for any display-based application is 60 Hz to ensure a smooth visualization for the user. The HRM provides a feature to measure the current frame rate [22]. Therefore, the refresh rate was simply obtained by checking the according value when tracking the QR code and visualizing the US volume. In the prestudy, participants mentioned latency issues only with 3D US. Accordingly, these two measurements were only conducted for this modality, and 2D US can be assumed to have equal or lower latency.

## 3. Results

### 3.1. Qualitative Prestudy

The qualitative prestudy included eight participants ($\mu_{age}=31.9,\sum_{age}=4.3$, five were female, three male). Six participants were physicians. Specializations included radiology, vascular surgery, anesthesiology, and internal medicine; all but one were residents. The other two participants were medical students close to finishing their studies and with minor clinical experience. Except for the two medical students, all participants regularly performed sonography (either diagnostically or interventionally), with four of them performing this routinely. All but one participant had no prior experience with AR.

**Figure 7:**
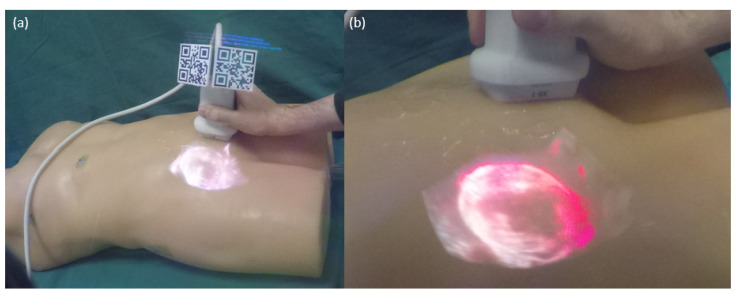
Photographs taken through a lens of HoloLens 2. Note that this results in worse overlay accuracy and color-shifting compared to wearing the HMD. (**a**) US probe including QR codes and the in situ visualized virtual volume of the bladder. (**b**) Close-up photograph of the volume.

Figure 7 and Video S3 illustrate the HoloLens user’s view of the imaged bladder. We identified six major categories outlined in Table 2, with the examples translated from German to English. Each category was also divided into subcategories, e.g., ’Benefit—Manual Coordination’, ’Use Case—Biopsies’, or ’Limitation—Tracking Unwieldy’. The entirety of coded segments can be accessed in the Supplementary Materials Table S4: Coded segments; the summary of each participant is available in the Supplementary Materials Document S5: Case summaries.

### 3.2. Quantitative Main Study

The experiment included 18 participants (n=18), of whom five were physicians with varying specializations and degrees of experience as well as one medical student. The remaining participants were engineers with previous experience working with sonography.

**Figure 8:**
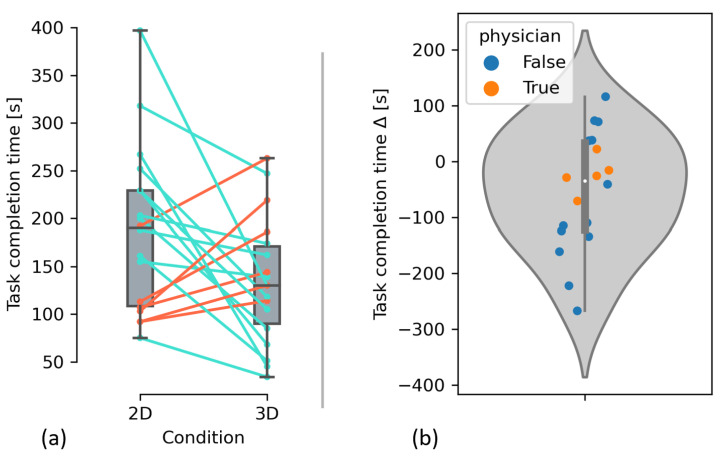
(**a**) Box plots and dot plots showing task completion time; lines connect the data points that belong to an individual participant. (**b**) Violin plot and dot plot showing the difference in task completion time. Negative values indicate less required time with 3D US and the HMD.

Participants completed the task significantly faster when using 3D AR compared to 2D AR ($\mu_{2D}=187.4$ s, $\mu_{3D}=134.1$ s, $\mu_{\Delta }=-53.3$ s, $\sum_{\Delta }=105.6$ s), with a relative decrease in task completion time of 28.4%. As the intraparticipant differences in task completion time sufficiently followed a normal distribution (Figure 8b), the hypothesis was tested with a paired t-test, and the significance level of 5% was adjusted for multiple testing ($H_{1}:\mu_{3D}<\mu_{2D}\approx 2.36\%<2.5\%$).

**Figure 9:**
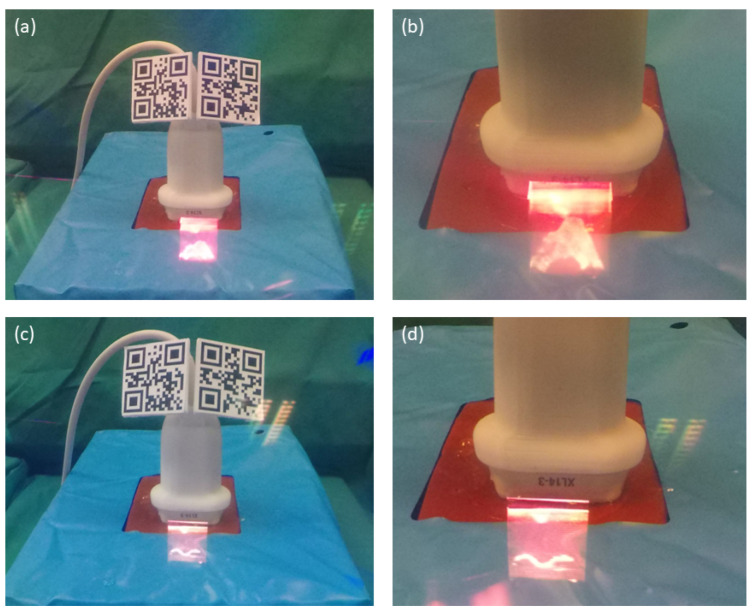
Photographs taken through a lens of HoloLens 2. (**a**) US probe including QR codes and the in situ visualized virtual volume. (**b**) Close-up photography of the volume showing the beginning of the bifurcation. (**c**) US probe including QR codes and the in situ visualized virtual image. (**d**) Close-up photography of the cross-sectional image showing the beginning of the bifurcation.

To test our second hypothesis, i.e., that participants successfully hit the target structure more frequently with 3D AR than with 2D AR, we performed an exact McNemar test. However, the differences (Table 3) were not statistically significant (p≈28.91%>2.5%). Due to the unexpectedly small sample size and the correspondingly low statistical power, we chose not to test our secondary hypothesis, i.e., that performing the task with 3D US reduces the subjective workload (measured via TLX) compared to 2D US. Another reason for this decision was that participants noted that they felt some items to be irrelevant (e.g., temporal demand) and accordingly answered them somewhat arbitrarily. Because we did not include per-item weighing, this introduces further noise and in turn further lowers the power of a potential test. Figure 9 shows the HoloLens users’ perspective of the 2D and 3D US image in situ. The data for the analyses can be found in the Supplementary Materials S6: Results main study.

### 3.3. Technical Evaluation

Latency was measured at 49.90 ± 12.92 ms (mean ± standard deviation) with minimum and maximum values of 25 and 75 ms, respectively. The diagnostics tool provided by the HRM showed a constant frame rate of 60 Hz while tracking the QR codes and rendering the streamed US volumes.

## 4. Discussion

Our results show that in situ overlays of real-time 3D US images can offer benefits over 2D images for US-guided needle placements, i.e., a reduction in task completion time. It appears, though, that this may depend on several conditions, i.e., when viewing US through an AR-HMD, 3D images are not, per se, superior to 2D images. Particularly, it must be ensured that target structures can be clearly distinguished from noise or occlusion—an issue not present with 2D US images due to their cross-sectional nature. This may be solved by choosing an appropriate use-case/anatomical structure, through fine-tuned volumetric rendering or potentially through additional segmentation and tracking within the US volume. We argue that all approaches are promising topics of research that can further evaluate how, and in which contexts, real-time 3D US can be advantageously utilized via an AR-HMD. Our qualitative results also support notions from previous publications that AR visualization of live US images can improve spatial understanding and reasoning as well as facilitate hand–eye coordination. The latter appears to be the case even when images are not spatially overlaid and instead ’float’ above the probe, i.e., when hands and US images can be viewed simultaneously. This is relevant because removing the need for accurately tracking the probe can reduce the technical complexity considerably. This also implies that AR visualizations for US are particularly useful for challenging image-guided tasks, e.g., needle placements, rather than routine or purely diagnostic use-cases. Regarding spatial understanding, our proposed setup does not only alleviates the need for mentally transforming 2D US images into 3D anatomical structures—viewing 3D US images also enables a more three-dimensional impression (Figure 7), given sufficiently echogenic structures.

In cases where even a sophisticated volume-rendering technique fails to adequately visualize the target anatomy or medical instruments, it is worth investigating the display of the three orthogonal slices as an alternative. This approach was also suggested by a participant in our prestudy who routinely performs ultrasound-guided procedures. Combined with an automatic detection of the needle and visualizing the according slice, as shown in [23], one can utilize the advantages of 2D and 3D US simultaneously.

Though we consider our results to show the general potential and properties of visualizing 3D US via AR HMDs, we caution against generalizing them into clinical contexts due to various limitations outlined below.

### 4.1. Limitations

For both the prestudy and the main study, a potential bias due to a novelty effect or social desirability cannot be excluded. We attempted to minimize these effects by comparing only AR-HMD conditions and not explicitly mentioning what we considered as the technical innovation. In addition, the qualitative study consisting of the interviews and data analysis was conducted by a person who was not involved in the technical development and is therefore less prone to bias the results.

#### 4.1.1. Prestudy

The sonography setup of the experiment was different from those typically used for clinical diagnostics: As opposed to sitting on a chair, with the US system located towards the head of the patient, participants had to stand (due to the height of the dummy lying on the experiment’s table) with the US system located at the dummy’s feet. Multiple participants remarked that this was slightly confusing. As a result, we changed our arrangement for the main study accordingly, and we suggest that, even for studies in a lab setting, arrangements should attempt to accurately reflect those found in the field.

The anatomy of the dummy (location and echogenicity of anatomical structures) was repeatedly mentioned to be surprising or confusing to participants. Furthermore, the tracking via the QR marker was an unwieldy addition to an already large 3D US probe. It also required users to move the probe in a way that keeps the QR marker visible. All of these factors were likely an unwanted distraction throughout the task. Investigating other probe tracking approaches, e.g., using retroreflective spheres, as described in [11], would be an important step to further develop this system.

#### 4.1.2. Main Study

The quantitative main study consisted of a relatively small sample size of 18. Additionally, only five participants were doctors, the intended user group of our system. Accordingly, our results cannot be generalized to doctors who perform US-guided tasks on a regular basis. Both aspects were due to difficulties in acquiring a sufficient number of volunteering doctors and time constraints.

Task completion times were relatively high when considering previous studies (mean completion times: 66.4 s [3]; 13 s [1]; 116.9 s [2]). An actual comparison is difficult as the tasks and previous familiarization differ between each study. Exemplarily, the participants in [1] performed not only one but ten punctures in a vessel phantom. Further, it remains unclear if the orientation phase was part of the measurement. We showed that the task completion time was significantly reduced with 3D US and the HMD. However, we want to emphasize that this is only valid for our lab scenario, which does not accurately reflect a vascular puncture on a real patient.

The power of our dataset regarding the second hypothesis (improved accuracy when using 3D AR compared to 2D AR) was low due to a small sample but also because of the binary nature of the measured variable. However, even though a continuous variable (e.g., distance to a target) may offer a more granular and statistically robust measure of accuracy, it may also significantly complicate the technical complexity by requiring a highly accurate tracking modality.

The success rates of 50% and 72% for 2D and 3D US, respectively, appear to be relatively low in this setup, which might be due to the fact that the majority of the participants were engineers. However, we assume that another major influencing factor is the limitations of the phantom used. The main purpose of the fabricated phantoms was to generate a realistic US-capable test subject, while avoiding animal experiments. Even though the chosen materials feature important characteristics such as a repair function and realistic acoustic attenuation and backscattering, we experienced several drawbacks during the main study. First, the perceived haptic feedback during needle insertion was different compared to human tissue. Especially, physicians mentioned that more force was necessary to insert the needle. Moreover, the wall-less structure of the dummy vessel did not provide any feedback once the lumen was reached, whereas in a real case, physicians may feel the perforation of the vessel wall. Additionally, blood will discharge from the external end of the needle once successfully placed in an artery, providing important feedback. However, the pulsatile pump of the vi-box could not provide sufficient pressure to do so in our setup. Lastly, the visibility of the needle in the US image and volume was challenging, which led to a limited visual guidance during the task. This restricted visibility of the needle may be due to the ballistic gel surrounding the needle, in addition to the probe, which, although a state-of-the-art linear 3D probe, does not provide the same image quality as a linear 2D probe.

Based on the feedback of the participants, the vessel structure including bifurcation was easily identifiable in the rendered US volume. Indeed, our volume rendering approach might be insufficient in a real patient as the vessel is surrounded by more hyperechogenic tissues such as different muscle layers. This is an important factor because differentiating between target and occluding structures was identified as a major difficulty in our prestudy. Accordingly, this phantom can be considered as a best-case scenario for our approach. As this is a study in a lab setting with a small sample size, we argue that it is acceptable to have best-case conditions to establish a theoretical base for more complex and realistic experiments in the future. Accordingly, we encourage future work to further investigate how real-time US volume renderings could more clearly visualize structures and instruments.

#### 4.1.3. Technical Evaluation

Our latency measurements show that the system introduces only a slight delay in visualizing the US volumes. However, the measured timespan does not reflect the time from image generation until image visualization but just the additional delay compared to the normal visualization on the US station screen. Still, we believe that a mean latency < 50 ms is a satisfactory result, also due to the fact that 50 ms is considered to be the threshold above which user experience seems to degrade in some of the most time-critical games [24].

The diagnostics tool of the HRM mode showed that our application runs at the target frame rate of 60 Hz, which confirmed the initial assumption that the HRM provides sufficient computing power for QR code tracking and volume rendering. A standalone application running solely on HoloLens 2 would be generally preferable but is currently not possible due to the small size of the HMD and the therefore limited hardware capabilities.

In this work, we did not perform a technical overlay accuracy test. Even though we think this is an important evaluation criterion, we argue that sub-millimeter accuracy is not crucial for this application as (1) the in situ visualization is just an indication of the actual target anatomy position, and (2) the needle and the target anatomy are visualized in the same coordinate system (US volume or image), reducing the required tracking and registration accuracy. To be more precise, even if the in situ visualization error leads to slightly wrong initial insertion of the needle, the interventionist can compensate for this once the needle becomes visible in the US volume in which the target anatomy is also visible. According to [10], a QR code’s position may drift up to ±2.5 mm during continuous detection, while the probe calibration error was quantified to 3 mm [14], leading to maximum error of around 5 mm.

## 5. Conclusions

Our work shows the feasibility of a novel system that visualizes real-time 3D US data via an AR HMD with minimal latency and a satisfactory frame rate. Furthermore, our experiments show that this may offer additional benefits in US-guided tasks (i.e., reduced task completion time) over 2D US images viewed in AR. We could also reproduce the results from the previous related literature that in situ visualization of US with AR can positively influence spatial understanding. At the same time, difficulties in distinguishing relevant structures from noise and occlusion were identified as a major limitation of this technology. However, when target structures are well distinguishable, AR HMDs offer a vivid spatial visualization of real-time 3D US. As a result, we argue that future research should aim to overcome the issue of visual occlusion so that this promising technology may be tested in more practical contexts.

## Figures and Tables

**Table 1:** The US station settings used for the prestudy (probe X6-1) and the main study (probe XL14-3).

	X6-1		XL14-3	
	2D	3D	2D	3D
Depth [cm]	Variable	Variable	4.5	4.5
FOV (azimuth × elevation)	Variable	Variable	NA	0° × 90°
Gain [dB]	0	0	0	0
Image brightness	Variable	Variable	25	35
$L_{l};L_{u};a$	NA	0; 255; 0.40	NA	0; 255; 0.14
Refresh rate [Hz]	Variable	Variable	8	8

**Table 2:** Main categories identified in the qualitative content analysis, including definitions, examples, and summaries per category.

Category	Definition	Example	Summary
**Benefit**	Segments mentioning a positive/helpful aspect of viewing ultrasound in AR (both 2D and 3D).	‘I liked it better with the glasses [HMD] simply because the image was larger […]’ (Participant 6) ’You basically see how the image changes, directly, without having to somehow reconstruct and think about the 3D from the screen’ (Participant 5)	The main benefits that were noted were that AR allowed for better manual coordination (both with the stationary and tracked overlay) and spatial understanding, often noting the ‘tomographic’ and ‘scrolling’ impression AR offers. Other benefits were that the AR image could be visualized larger and positioned freely.
**Familiarity**	Coded when voicing unfamiliarity, being used to something different, or mentioning familiarization with our system.	‘Yes, I’m looking at the stationary image up here because then I can move the ultrasound probe like I’m used to.’ (Participant 1) ’Right, now this is tricky. Because of course now you really only see the 3D image, right? Hm, that takes some getting used to, just because you don’t do that often.’ (Participant 7)	Many participants mentioned that they were unfamiliar with 3D sonography and its issues of occlusion and noise. Many also said they were used to a stationary image at the side (often in context of looking at or preferring the stationary AR image, rather than the tracked view.) The majority opined that getting used to this technology is only a matter of practice and not a major hurdle. Additionally, some radiologists mentioned that they are used to viewing volumetric images and framed this as learning to view just another imaging modality.
**Limitation**	Segments describing negative aspects, expressing frustration or not being able to do things in a certain way.	‘Okay, it’s delayed again here’ (Participant 3) ’Well you can’t just go through your motions like that. This stupid QR code has to be visible somehow.’ (Participant 4)	All participants either struggled with or criticized the tracking of the probe as being unwieldy. Many noted that they found the volumetric visualization unintelligible, some describing it as “tiny clouds” in front of the organs. Several participants were confused by or commented on the unusual anatomy of the dummy (e.g., the position of the right kidney.) Some also noted latency issues in the 3D mode.
**Preference**	Statements indicating preference for a type of visualization, e.g., conventional or 3D AR.	‘And that was a bit easier without the glasses [HMD] in the 3D mode’ (Participant 2) ’Difference from Augmented to screen is relatively big and Augmented is just much more user-friendly because you could orient yourself.’ (Participant 5)	Half of the participants noted preference for the floating mode, often in context of being used to a stationary image away from the situs. Other than that, preferences seemed to vary and were often tied to specific issues such as tracking issues, volumetric readability or spatial understanding.
**Use Case**	Coded when participants suggest or speculate on use cases/applications of our setup.	‘I actually think if you’re getting started with sonography it’s going to be much easier if you’re doing it with the glasses […]’ (Participant 4) ‘Generally I feel like this is for needle placements, like I said, with the HoloLens, if you look at it and insert the needle where you’re looking, that is better than looking at a screen.’ (Participant 7)	By far the most commonly mentioned use-cases were image-guided procedures, particularly needle placements/biopsies. This not only occurred in the interview when being specifically asked about biopsies as a use-case but also occurred spontaneously (e.g., during the think-aloud phase.) Some participants noted that it would be particularly useful for complex and elective procedures rather than routine tasks with low accuracy requirements. Another commonly suggested use case was for beginners learning the basics of sonography. Others noted situations where the AR-HMD could replace screens to make more room, e.g., crowded operating or trauma rooms.
**Verdict**	Judgments about the technology in its entirety, particularly regarding whether it is useful or practical. If specific aspects are mentioned, either positively or negatively, they are coded as ‘Benefit’ or ‘Limitation’, accordingly.	‘Yeah, well, somehow this 3D thing isn’t really useful for me I’d say.’ (Participant 4) ‘But that’s pretty sexy, having theultrasound image directly on top of the body somehow, like, overlaid.’ (Participant 6)	Verdicts were often unspecific and either positive (e.g., calling the technology ‘awesome’ or ‘pretty’) or negative, mentioning difficulties with AR, particularly the volumetric view. Several participants also mentioned that this setup may be unnecessary or not helpful, particularly in diagnostic contexts. Some also mentioned that they have learned ultrasound-guided procedures without AR and perform them sufficiently well, i.e., they do not see a need for additional technology.

**Table 3:** Results of the vascular puncture attempts.

	2D US with HMD	3D US with HMD
**Successful puncture**	9	13
**Unsuccessful puncture**	9	5

## Data Availability

Not applicable.

## Supplementary Materials

The following supporting information can be downloaded at: https://www.mdpi.com/article/10.3390/s23042168/s1, Document S1: Prestudy protocol; Python script S2: evaluation code; Video S3: 3D ultrasound in situ visualization of the bladder; Table S4: Coded segments; Document S5: Case summaries; Table S6: Results main study.
