# Supplementary material for: Augmenting Image-Guided Procedures through In Situ Visualization of 3D Ultrasound via a Head-Mounted Display
Source: Sensors (Basel). 2023 Feb 15;23(4):2168. doi: 10.3390/s23042168 (PMC9961663; doi:10.3390/s23042168)
Supplement: Supplementary file 1 [file sensors-23-02168-s001.zip › Document S1.PreStudyProtocol.pdf]

Protocol – qualitative pre-study

General Agenda

- Introduction
- Instructing participant
- User test, including think-aloud session
- Semi-structured interview
- End of experiment

Individual Steps

Introduction

- Welcoming participants and introducing study personnel
- Thanking participant
- Explaining context of this research project
- Agenda of the experiment
- Data privacy (purpose of data collection, further processing of data, participant's data privacy rights)
- Time for questions/clarifications
- Consent (once per form)

Instructing participant

- Explain augmented reality and head-mounted displays
- User instructions for HoloLens 2, also regarding our ultrasound application
- Explaining 'thinking aloud'

User test

- If unfamiliar with 3D ultrasound: test run with (conventional) 3D ultrasound
- **Start of audio recordings**
- Freely testing technology on dummy, including thinking aloud
- Ends either when participant finishes by themselves or after 5 minutes of testing

Semistructured interview

See below for the interview field manual

End of experiment

- Thank you (for participating)
- Informing them about the next steps of the project, asking whether we may contact them for the following quantitative study

- Goodbye

Interview field manual

Personal data

- Age
- Specialization, Years of Experience
- Previous use of ultrasound: use cases and degree of experience
- Pre-existing experience with AR/XR

Getting started

- How unfamiliar did it feel to use the AR application?

Differences

- How did the AR application affect the difficulty of the task? Was there a difference between 2D and 3D AR?
- Did the AR application noticeably influence how you perceived the US images and how you approached the task?
    - If so, how? If not, why not?
- If not previously mentioned – how/to which degree did the AR application have an impact on your spatial understanding of the anatomical structures?
- If you compare the visualizations of the 3D US images – do you prefer one modality of visualization and if so, why?

Practical Applicability

- Could you imagine using this AR application in your medical work?
    - If so, for which tasks?
        - Which effects would this have on the task, in which advantages and disadvantages would this result?
        - Does the additional technical complexity of the AR setup justify the expected benefits?
        - How would you see this in the context of vascular punctures?
    - If not, for which reasons?
- How often did you look at the conventional US monitor when using the AR app and when not using it?
    - If you switched gaze/contexts less with AR – would you expect any benefits during clinical practice from this?

Concluding verdict

- Would you like to use this AR application at work?
    - When and for which reasons, respectively when and why not?
