# Supplementary material for: Augmenting Image-Guided Procedures through In Situ Visualization of 3D Ultrasound via a Head-Mounted Display
Source: Sensors (Basel). 2023 Feb 15;23(4):2168. doi: 10.3390/s23042168 (PMC9961663; doi:10.3390/s23042168)
Supplement: Supplementary file 1 [file sensors-23-02168-s001.zip › Document S5.CaseSummaries.pdf]

Case Summaries

Participant 1

Participant 1 (P1) is a female radiologist in her mid-thirties at the end of her residency. She also has surgical experience and frequently performs sonography.

During 2D ultrasound she used mostly the stationary view, noting that this is due to her being most accustomed to this type of view. P1 noted that the tracking was unwieldy, which made it difficult to replicate the standardized sonography view planes/angles that she is used to. She noted latency, saying she moved faster than the volumetric ultrasound view would update its position. Another issue was that she often could only see parts of the AR image, saying that she would need to view it from further away, but her arms were too short. She also pointed out that 3D ultrasound was difficult to read and unfamiliar, but that both 3D and 2D AR would be easy to get into with practice. According to P3, this may also depend on users and their specializations, noting that radiologists are used to seeing (3D) tomographic images. She concluded that this setup would be most beneficial in image-guided interventions when it would be possible to see the instruments, site and ultrasound image simultaneously while also being able to place the image at any convenient position in the room without relying on a screen. However, P1 considered the technology to be of little use in purely diagnostic contexts.

Participant 2

Participant 2 (P2) is a female medical student in her fifth year. She is in her mid-twenties with very limited ultrasound experience and no prior exposure to AR.

Though initially impressed by the technology, she struggled with the tracking and also noted latency issues in the 3D mode. P2 also noted that the overlay visualization obstructed her view of the patient, which she used to orient herself. P2 said she was having difficulties understanding the volumetric rendering, describing it as looking like “small clouds”, which she found even more difficult with the HMD. However, she noted that in 2D, she liked being able to freely position the image and also at a larger scale. Additionally p2 mentioned that she thinks that it might take some practice, but she found it easy to adapt to the AR visualization, noting that if she had more practice she would probably prefer the 2D AR visualization.

Participant 3

Participant 3 (P3) is a female medical student in her last (practical) year. She is in her early thirties with very limited ultrasound experience and no prior exposure to AR.

P3 struggled with the tracking, especially on the side of the patient that is opposite of her. She also noted latency issues with 3D, as well as anomalies in the dummy’s anatomy. She also mentioned that the AR image occluded the patient. When testing different US frequency settings, she noted that the

image was getting smaller at higher frequencies, which made sense to her but was initially unexpected as images get larger with higher frequencies on a conventional monitor. P3 also noted the issue of requiring additional technology, which adds another potential point of failure during clinical work. According to P3, understanding the volume was difficult due to obstructing elements, especially because she lacked experience with 3D US, though she preferred the AR visualization. She also found the AR HMD to offer better depth perception and noted that seeing the 2D image in AR is like seeing the body 'sliced open'. She found the latter to improve spatial understanding and manual coordination, which she imaged to be useful especially for beginners but also during biopsies. However, in her final verdict she thought that the technology was currently more of a toy, noting that US requires experience and that AR HMDs likely are not as useful to experienced users anymore. Still, she would be interested in testing it out and seeing how the technology performs in practice.

Participant 4

Participant 4 (P4) is in the last year of her residency in a department of internal medicine. She is in her early thirties. P4 has spent almost a full year focusing on diagnostic sonography but also performs ultrasound-guided biopsies. She has not used XR previously.

P4 noted that the arrangement of the setup with the sonography station to her left was unfamiliar. She also had issues with the tracking and was occasionally frustrated by it. Like P1, she noted that viewing the image in AR in a stationary position felt familiar. P3 noted that the dummy anatomy had several surprising aspects. She found 3D ultrasound to be hard to understand, as well, though noted that, in AR, it "looks nice" (particularly the bladder) and is likely easier to understand due to the additional depth offered by the HMD. P3 noted that the technology would be unnecessary when performing diagnostic sonography, but instead could be beneficial during ultrasound-guided procedures. In this regard, she particularly noted the ability to view both the instrument and the ultrasound image simultaneously, even if the image is not tracked. She also suggested that the AR visualization might help with accuracy during biopsies.

Participant 5

Participant 5 (P5) is a male radiologist in his first year of residency with previous experience at a surgical department. He utilizes ultrasound for various diagnostic purposes. P5 is in his early thirties and has no previous experience with XR.

Though P5 also noted difficulties with the tracking, he moved frequently to adapt to the viewing angle for the QR code. He also noted that the glasses were 'many layers of glass or plastic' to be worn, though when later asked about it he answered that it was still acceptable. P5 mentioned difficulties in understanding the 3D image and finding anatomical structures with it, though noting that it may be due to his lack of experience with this imaging modality. He also noted that the ultrasound dummy had various unexpected properties, including an untypically echogenic bladder, which was also easy to see in volumetric AR. P5 noted several benefits of the technology: First, he mentioned that it is convenient to be able to freely place the image, which may be beneficial in rooms filled with other devices or people, like a trauma room. He also speculated that with progressing technology, it may be feasible to carry only an ultrasound probe which could directly

connect to a HMD and drastically decrease the size and improve the mobility of ultrasound technology. Additionally, P5 explicitly mentioned better manual coordination with the AR HMD as a benefit. He also noted improved spatial understanding, saying that the tracked position is like 'slicing through' and that it removes the mental transformation to map the 2D image from the monitor into the real world in 3D.

Participant 6

Participant 6 (P6) is a male radiologist in third fourth year of residency. He is in his early thirties and performs sonography both for diagnostic and interventional purposes such as vascular punctures. P5 has no prior experience with XR, other than 'just playing a bit of Pokémon Go'.

P6 did not mention major difficulties with the optical tracking of the probe, only mentioning that it might interfere with certain procedures that require mobility, but expecting the optical tracker to be a temporary solution and to improve over time. However, he found the volumetric visualization to be hard to understand, citing a lack of familiarity with 3D US. An exception to this was the bladder, which he mentioned was very easy to see in 3D He noted that 3D US is likely just another imaging modality to learn, e.g. understanding how certain anatomical landmarks look like in this technology. He also noted that radiologists already tend to form a 3D image of structures when performing 2D ultrasound, likely due to seeing many tomographic 3D images as slices at work. P5 noted that this technology may be useful for beginners to understand the spatial alignment of the ultrasound probe, as well as during ultrasound-guided procedures by improving accuracy and being able to see simultaneously see the image and the needle. He imagined that this would be especially the case for elective procedures where additional technology poses no issue; it may even replace CT in some image-guided procedures to reduce radiation exposure and to free CT resources. Despite listing the use cases and expressing interest in the technology, he noted that he would likely not use the technology as he has learned to perform ultrasound-guided procedures without it. Additionally, P5 mentioned that the biopsies he performs usually do not require an accuracy of a few milimeters and as a result, he does not need to focus on his hands or the needle while performing them.

Participant 7

Participant 7 (P7) is a male consultant/attending surgeon in his late thirties. His ultrasound experience is mainly in the context of image-guided procedures. He has used XR technology in other research projects.

P7 only had minor issues with the tracking of the ultrasound probe, calling it 'unfamiliar'. However, he too had difficulties interpreting the 3D/volumetric ultrasound images and finding anatomical structures with it (except for the bladder), noting he does not use 3D US frequently. He suggested that it may be easier to view only three image planes (a view offered by the ultrasound system) and that this would likely be better for his use cases. P7 also noted that the dummy's anatomy has certain unexpected aspects, like the position of the right kidney. P7 could imagine that the technology may offer benefits for biopsies, especially for complex/difficult cases. He explains that it would be beneficial to see the image projected onto the body and being able to position the needle accordingly. However, he doubts that the technology would offer benefits for diagnostic purposes.

Participant 8

Participant 8 (P8) is a female anaesthesiologist in the last year of her residency. She uses ultrasound primarily for regional anaesthesiology but also has experience with diagnostic sonography. Other than having tested a VR HMD once she does not have any previous experience with XR.

P8 said that the technology was ‘cool, really cool’, comparing it to ‘scrolling through’ the body (with 2D US in AR). She also noted that the impression is very three dimensional while viewing the bladder with 3D US in AR, offering ‘additional perspectives’ into the body which you do not get with conventional ultrasound systems. However, she also noted difficulties in interpreting the volumetric image, noting additional artifacts in 3D as well as a lower perceived range of grayscale values which are important for interpreting ultrasound images. P8 preferred the AR visualization, calling the visualization on the regular monitor ‘horrible’, where as AR offered better orientation and readability, which made the user experience considerably better than the monitor, according to P8. She could imagine that the technology could be useful for ultrasound-guided needle placements, such as central venuous catheters. She noted that this would be particularly the case with an out-of-plane technique, because with an in-plane technique one would constantly look at the screen. With out-of-plane techniques, though, it is important to see most of the surrounding anatomy, but also the needle, which is not possible with conventional 2D but may be feasible with 3D US AR.
